# A Global Multi-Source Tropical Cyclone Precipitation (MSTCP) Dataset

**DOI:** 10.1038/s41597-024-03395-w

**Published:** 2024-06-11

**Authors:** Gabriel Morin, Mathieu Boudreault, Jorge L. García-Franco

**Affiliations:** 1 Department of Mathematics, Université du Québec à Montréal, Montreal, Canada; 2 Lamont-Doherty Earth Observatory, Columbia University, New York, USA; 3 Escuela Nacional de Ciencias de la Tierra, Universidad Nacional Autónoma de México, Mexico City, Mexico

**Keywords:** Hydrology, Atmospheric dynamics

## Abstract

A global tropical cyclone precipitation dataset covering the period from January 1979 to February 2023 is presented. Global precipitation estimates were taken from the newly developed high-resolution Multi-Source Weighted-Ensemble Precipitation, version 2 (MSWEP V2) and TC tracks were obtained from the International Best Track Archive for Climate Stewardship (IBTrACS) dataset. This Global Multi-Source Tropical Cyclone Precipitation (MSTCP) dataset is comprised of two main products and files in the format of tables: the main and profile datasets. The main file provides various TCP statistics per TC track, including mean and maximum precipitation rates over a fixed and symmetrical radius of 500 km. The profile dataset comprises the azimuthally averaged precipitation every 10-km away from the center of each storm (until 500 km). The case study of Hurricane Harvey is used to show that MSWEP estimates agree well with another commonly used satellite product. The main statistics of the dataset are analyzed as well, including the differences in the dataset metrics for each of the six TC basins and for each Saffir-Simpson category for storm intensity.

## Background & Summary

Heavy rainfall from landfalling tropical cyclones (TCs) causes devastating impacts such as loss of life, damages on property, infrastructure and supply chains^1–3^. These flood-related hazards from TCP are exacerbated by local vulnerabilities^4^ and by the occurrence of other extreme weather events, also known as compound events^5^. TCP is not only a hazard but also an important contributor to total rainfall, and therefore water supply, since TCs can account for more than 35% of the annual rainfall over Australia, Taiwan, the Philippines and Mexico^6,7^.

Reliable observations of TCP are therefore paramount to understand the role of TCs in local and global water budgets, and the relationship between TCP, floods and societal impacts. Observational estimates of TCP are essential for studies seeking to understand its environmental controls^8^, its spatial distribution and contribution to the total annual rainfall and extreme precipitation^9^, trends in TCP^10,11^, the response to radiative forcing^12^, as well as model evaluation^13–15^.

TCP estimates are obtained from gridded-station data, aircraft *in-situ* data, reanalysis or satellite retrievals. Gridded-station data provide observations of TCP that can extend as far back as the early 1900s^10,16^. However, high-density station data with long coverage can only be found in a few countries and regions. Aircraft radar data is even more limited as only a small number of cases have been sampled in recent decades^17^. Reanalysis data has reasonable spatial and temporal coverage but precipitation is simulated by the driving forecast model and reanalyses have a higher discrepancy in their representation of TCP than in total precipitation^18^.

Satellite precipitation retrievals are therefore the best option for continuous and reliable monitoring and research of global TCP^19^. For instance, the Tropical Rainfall Measurement Mission (TRMM) Multisatellite Precipitation Analysis (TMPA) dataset, launched in 1997, has been extensively used as benchmark of total precipitation in the tropics and TCP due to its relatively high resolution (0.25°) and temporal (3-h) resolution^20^. TCP was estimated in TRMM by dozens of studies using similar methodologies^7,9,21^. However, the satellite was discontinued in 2015 and the dataset ends in 2019^22^.

The Multi-Source Weighted-Ensemble Precipitation version 2 (MSWEP V2) is a global gridded precipitation dataset spanning 1979–present^23^. Relative to former precipitation products such as TRMM, MSWEP has better temporal coverage (starting in 1979), higher horizontal resolution (0.1°) and a similarly high temporal (3 hourly) resolution. Due to these advantages, MSWEP has recently been used as a primary source of information for assessments of mean and extreme precipitation^24,25^. In fact, several studies have already analyzed TCP using MSWEP^26–28^ but the resulting datasets are not directly available for download, or have a limited temporal or spatial coverage.

The estimation of TCP requires a relatively costly computation. Firstly, a track, most frequently obtained from the International Best Track Archive for Climate Stewardship (IBTrACS), is used to transform the coordinate system of the gridded precipitation dataset into a cylindrical storm-relative system with the coordinates being the radius from storm-centre and their angle relative to the north or to the vertical wind shear vector. After this coordinate transformation, various metrics can be computed such as area-weighted averages of precipitation, calculations of the radius of maximum rain-rates (RMR), the azimuthally averaged structure of precipitation and estimates of the rainfall area (RA)^9,11,13,14,29^. These metrics are calculated for each track point, i.e., each TC observed every 3 or 6 hours. Therefore, processing TCP on high resolution datasets for the full coverage of the dataset (>40 years) has a relatively high computational cost, particularly in the newer and higher resolution datasets such as MSWEP.

After the discontinuation of TRMM, it is likely that MSWEP V2 will become the standard for future TCP analyses. And as such, it is likely that many studies will then repeat the same basic methodology to diagnose TCP in MSWEP. In this context, the Global Multi-Source Tropical Cyclone Precipitation (MSTCP) dataset is introduced to provide a global dataset of TCP metrics derived from MSWEP. The dataset contains various TCP statistics per storm track for all basins of the IBTrACS dataset since 1979 based on the MSWEP and IBTrACS datasets. In addition, the dataset contains the azimuthal average precipitation by 10-km steps away from the eye of each storm (until 500 km). The TCP metrics are provided in a user-friendly format and are considerably less extensive than the full MSWEP dataset.

## Methods

### Input data

Tropical cyclone track data is from the International Best Track Archive for Climate Stewardship (IBTrACS) version 4^30,31^. All IBTrACS entries are included from January 1979 until February 2023 from the six cyclogenesis basins: Atlantic (NA), Eastern North Pacific (EP), North Indian Ocean (NI), South Indian Ocean (SI), South Pacific (SP), Western Pacific (WP).

The Multi-Source Weighted-Ensemble Precipitation (MSWEP) version 2 dataset is a global precipitation product available at 3-hourly 0.1° from 1979 and onward with a global coverage over land and sea^23^. In total, MSWEP merges data from over 15 datasets including rain gauge, satellite and reanalysis data^23^. One relevant aspect of MSWEP is the inclusion of the period prior to the TRMM era, extending the record back to 1979. This extension was achieved by including gauge, reanalysis and infrared derived estimates of precipitation.

### Method

The core of the methodology lies on the intersection of each entry in the IBTrACS dataset (LAT, LON) with the corresponding time (ISO_TIME) and precipitation field in MSWEP. Intersections are computed from January 1, 1979 to February 14, 2023.

**Fig. 1:**
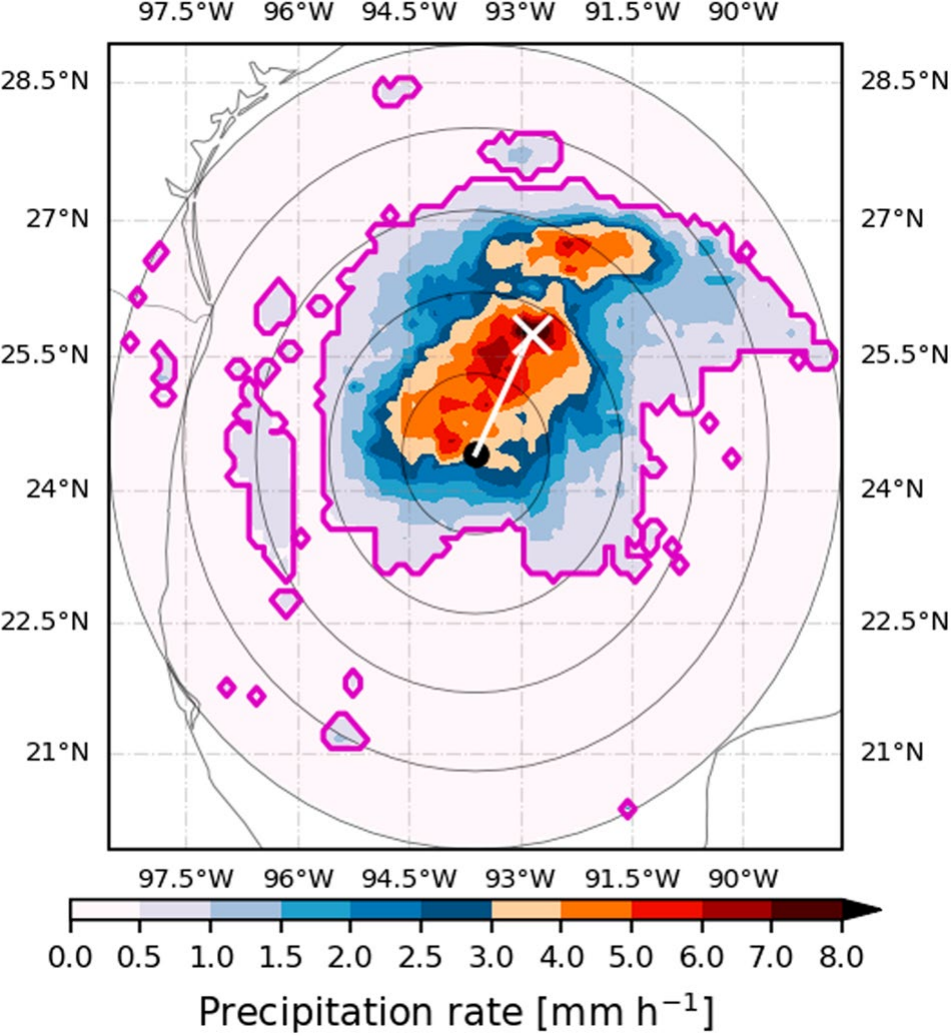
Illustration of the TCP metrics computed in the Global MSTCP dataset for Hurricane Harvey observed on August 24, 2017 at 18:00 (UTC). In the main dataset, the 500 km annuli (annotated as the outermost gray circle) is used to compute the mean precipitation. The white cross (X) denotes the location of maximum precipitation rates. The distance between the center and the white cross determines the radius of maximum rain. The binning of the data into radii increasing every 10 km is illustrated by annotating the 100, 200, 300 and 400 km radii. The rainfall area is shown as the magenta lines, as contours of the cells with at least 0.5 mm h^−1^.

The first analysis computes storm wide statistics using a 500 km radius geodesic buffer for each trajectory point. These metrics are the average TCP, maximum TCP rates, radius of maximum rain (RMR) and rainfall area. These values are stored in the *main* dataset. Figure 1 illustrates the main metrics of the dataset. The full black circle in the middle represents the estimated center of hurricane Harvey on August 24, 2017 at 18:00 (UTC). The outermost circle represents the 500 km radius, a commonly used threshold to define TCP^14,32^. This radius defines the circle where all statistics are computed. Precipitation outside this circle is ignored.

The area-averaged TCP is computed as the cumulative rain within that 500 km circle divided by the area of the circle^15,29^. The fixed 500-km threshold as a radius to diagnose the average TCP has been extensively used in the literature^1,7,15,33^. It was also shown that that the 500-km threshold is, on average, a good approximation to the area where TCs produce rainfall because this radius roughly matches the scale of a TC cloud shield^29,34^. However, other studies have analyzed TCP using a flexible threshold that is case-by-case dependent. For example, some authors used an object-based tracker and found that more detailed determination of TCP can lead to higher average precipitation rate estimates compared to fixed-threshold estimates^21^. The MSTCP dataset uses the fixed 500-km threshold since it is arguably the more commonly used approach.

The rain area (RA) is defined as the area (km^2^) within the circle where precipitation is above the threshold of 0.5 mm h^−1^, following previous studies^8,35,36^. The RA is estimated by adding the estimated area of each pixel with estimated precipitation rates higher than the threshold. This is illustrated in Fig. 1 as the magenta contour which in this case shows that most of the rainfall area is found within 300 km of the storm centre. Other studies have chosen smaller^37^ or larger thresholds^16,38^ for the rain-rates that define the RA which makes our choice an intermediate threshold for the RA.

In addition to the average precipitation rates, the MSTCP dataset provides the maximum precipitation rates, and their storm-relative location, for each snapshot derived from MSWEP. The extremes of the TCP distribution, as well as their spatial and temporal variability, are an active topic of research^6,39^ so the inclusion of this metric in the dataset is warranted. The maximum TCP reported in the dataset is the maximum value of precipitation rate found for each time-step. In the dataset, the location of the pixel of the maximum precipitation rate is also reported. In Fig. 1, the white X represents the location of the maximum precipitation rates (within the 500 km circle)^40^. The line between X and the storm centre is therefore the radius of maximum rain^37,40^.

In the second analysis, we compute azimuthally averaged statistics of TCP. This methodology aims to average out the variance of the precipitation that is unrelated to the radial coordinate and therefore only measures how precipitation changes as a function of distance from storm center. This choice means that the MSTCP dataset may not be suitable for studies on rainfall asymmetries in TCs. The dataset only provides the symmetric structure of precipitation given that it is a more commonly used diagnostic and also given the different ways to define rainfall asymmetries (storm-relative, shear-relative, land versus ocean).

To compute the azimuthal averages of precipitation, each pixel or grid-point was binned into 50 discrete non-overlapping geodesic ring buffers, or bins representing the distance to storm centre, with a 10 km spacing. Figure 1 illustrates this procedure by plotting equally distant (100 km) annuli from the storm, so that the calculation computes the average of all grid-points within each annulus. All raster cells whose centroids fall within the geodesic buffer or bin are used to compute the azimuthally averaged TCP rates^13,29,41^. The *profile* dataset then reports, for each entry in IBTrACS that is found in the main dataset, 50 pairs of data points which are the radial bin or buffer [km] and the corresponding average precipitation for that bin (see the following section for more detail on the output).

To mitigate the unequal grid cell area problem, statistics were area-weighted. Grid cell areas in km-sq were estimated using the *Climate Data Operators* gridarea operator^42^. Distance conversion from degrees to kilometers were computed with the Haversine formula with an Earth radius of 6371 km.

To manipulate the original MSWEP dataset, compute statistics, assemble and save both CSV files, the computation time required is approximately 200 hours on a 12-core/24-thread (AMD Ryzen 9 3900x) computer with 256 GB of RAM (4 × 32 GB DDR4) with a 4TB HDD (5400RPM SATA 6 Gb/s 64MB Cache (RoHS)) under Ubuntu 20.04.6 LTS.

## Data Records

The Global MSTCP dataset^43^ is freely available for download at https://zenodo.org/records/10105751 and can also be found with the 10.5281/zenodo.10105751. The MSTCP dataset^43^ is made of two CSV files: *main* and *profile* datasets. The columns for both files are detailed below. The *main* dataset comprises precipitation statistics presented in a format similar to IBTrACS, where each row corresponds to data per 3-hour segment. The *main* CSV file size is about 50 MB. The *profile* dataset comprises azimuthally averaged precipitation statistics in 10 km bins from storm center out to 500 km. Each row corresponds to a 3-hour segment with a given distance from the eye: 10, 20, …, 500 km. The *profile* CSV file size is about 1.3 GB.

The IBTrACS^31^ dataset is available at: https://www.ncei.noaa.gov/products/international-best-track-archive. The CMORPH^44^ dataset is also available from the Climate Prediction Center (CPC) at https://www.ncei.noaa.gov/products/climate-data-records/precipitation-cmorph.

## Technical Validation

### Hurricane Harvey

**Fig. 2:**
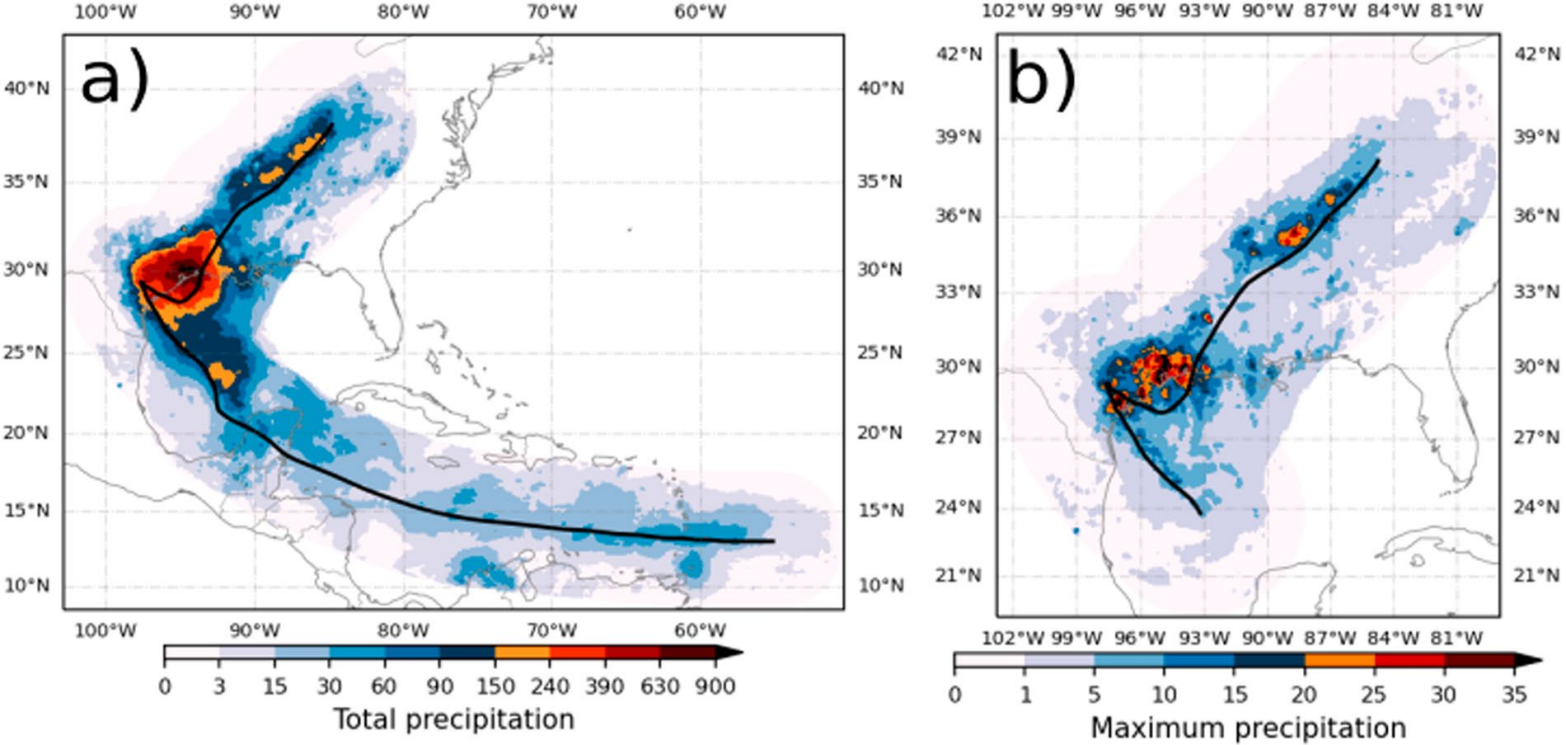
Hurricane Harvey (2017) estimated TCP in MSWEP. (**a**) Accumulated precipitation amounts [mm] and track (solid line). (**b**) Maximum hourly precipitation rates [mm h^−1^].

Hurricane Harvey flooded Houston and nearby areas in 2017 due to enormous rainfall amounts and precipitation rates. The floods associated with Hurricane Harvey were made more likely by greenhouse warming that increased the total amount of water vapour available in the atmosphere^45^ and more damaging by urbanization^46^. Fig. 2a shows the total amount of precipitation associated with Hurricane Harvey as estimated by our analysis of MSWEP. Most of the precipitation was observed during landfall in Texas, where the TC turned southeast for a short time followed by a northward trajectory into mainland United States. The stalling of Harvey around the area was devastating due to the very high rainfall rates (Fig. 2b) and the relatively long time Harvey was around the Houston area.

The spatial distribution and rainfall total amounts estimated by MSWEP agree well with those reported in previous studies using rain-gauge, radar and other satellite-derived products^47^. The spatial distribution of maximum precipitation rates with MSWEP (Fig. 2b) also agrees with the literature^47^. This demonstrates that our estimates of average TCP and maximum TCP are consistent with previous studies in this case.

**Fig. 3:**
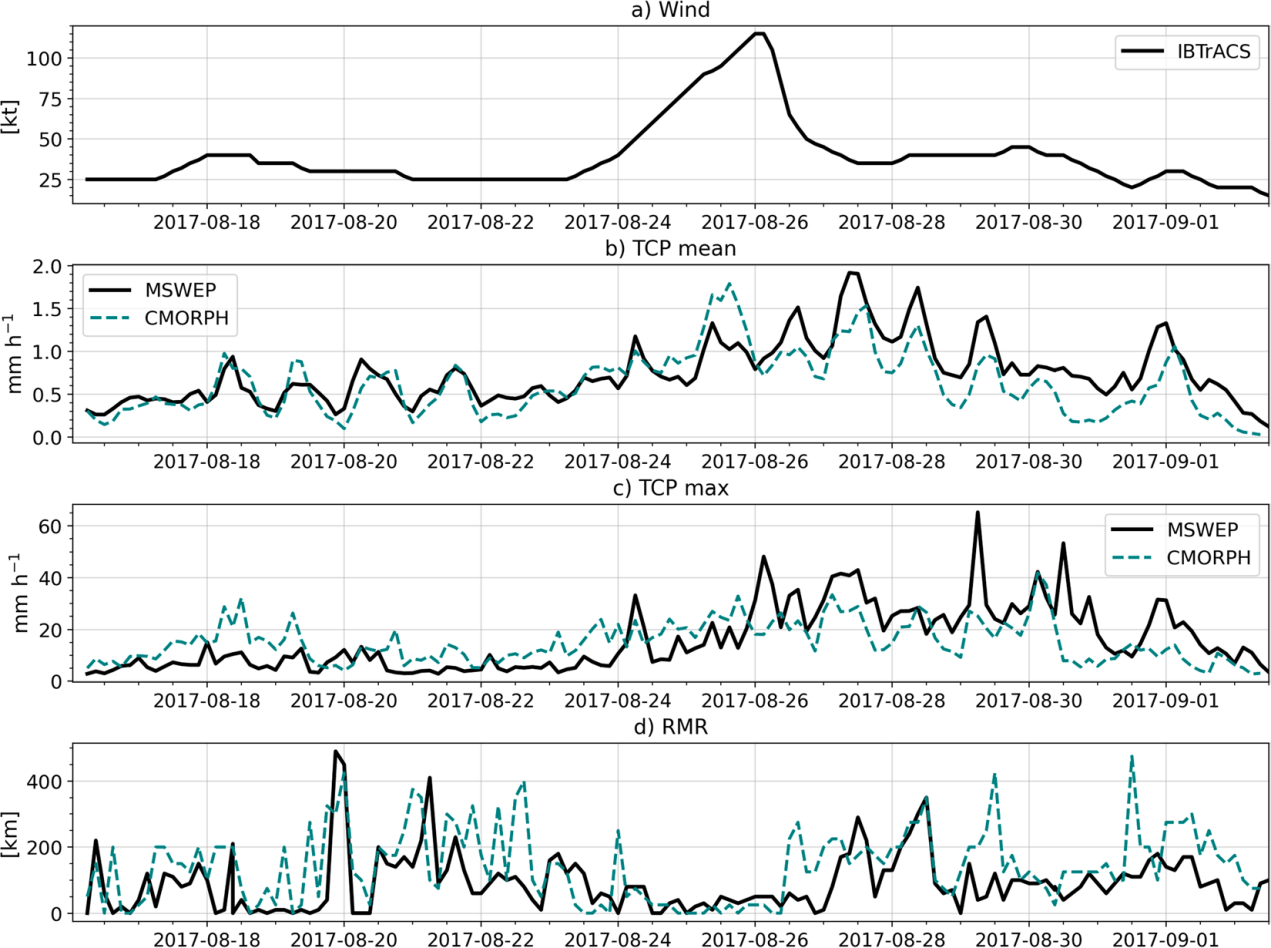
Time series of (**a**) maximum sustained wind speed [kt], (**b**) mean TCP, (**c**) maximum TCP [mm h^−1^] and (**d**) RMR [km] in Hurricane Harvey (2017) estimated from (**a**) IBTrACS and (**b-d**) CMORPH and MSWEP.

To compare the results obtained from MSWEP, we have repeated the analysis of Hurricane Harvey using the CMORPH dataset^44,48^. Fig. 3 compares the time series of the main metrics diagnosed with MSWEP for Hurricane Harvey (2017) with the estimates from CMORPH. The estimates of the average precipitation within 500 km of the center of the storm are very similar between the two datasets, with both datasets showing a clear daily cycle. In both datasets, mean precipitation increases after August 24, 2017, which is the period where Harvey began intensifying.

The maximum precipitation rate estimates (Fig. 3c), however, are slightly higher in MSWEP, especially during the period of highest intensity of Harvey. This may be due to the higher spatial resolution of MSWEP (0.1°) compared to CMORPH (0.25°). The RMR also appears to be a more noisy quantity (Fig. 3d) and the estimates from MSWEP sometimes differ notably from those of CMORPH. However, both datasets suggest a very low RMR at peak intensity which makes sense with a strong storm that has a compact circulation and strong ascent near the eyewall, likely linked to a short radius of maximum wind (RMW). Overall, our analysis shows that the estimates of TCP for Hurricane Harvey from MSWEP agree well with those of CMORPH. It should be noted that CMORPH and MSWEP share several input products in their merging algorithm so they are not independent datasets.

### Main dataset

The four metrics that compose the main dataset are the mean TCP, maximum TCP rates, RMR and the rainfall area (RA). In this analysis, we use the Saffir-Simpson scale to categorize storms as a function of their intensity. The IBTrACS definitions of basins are used as well to analyse inter-basin differences.

Table 1 reports the number of entries found in each TC basin across the dataset, as well as the average values of the chosen TCP metrics. The number of counts per basin shows that the dataset includes a considerable amount of cases which may prove useful to define climatologies conditioned on storm characteristics, basin or environmental conditions. Differences in the counts of TCs between basins in the dataset is due to the different number of observed TCs in each basin, as more TCs are observed in the WP and less TCs in the NI for example.

**Table 1:** Mean statistics separated per TC basin. Shown are total entries (counts) in the dataset, and the basin average of mean TCP (in mm h^−1^), max TCP (in mm h^−1^) and the RMR (in km).

BASIN	Count	TCP_*mean*_ [mm h^−1^]	TCP_*max*_ [mm h^−1^]	RMR [km]	RA [10^5^ km^2^]
NA	38277	0.78	12.8	94	2.72
EP	45309	0.68	11.3	92	2.42
NI	12844	0.93	14.3	95	3.28
SI	39249	0.97	12.8	113	3.33
SP	20896	1.18	14.3	117	3.64
WP	100364	1.18	13.8	100	3.94

Various studies have shown how these different TCP metrics vary spatially, due to environmental conditions^29^, and also due to storm scale processes^13,41^, that usually are associated with mean storm intensity. However, the TCP versus storm intensity relationship is highly non-linear in observations^10^.

Table 1 shows how each metric varies with TC basin. The average TCP in a 500 km radius is highest in the SP and WP and lowest in the EP on average which may be largely due to median storm intensity differences^29^. The maximum TCP rates are highest in the NI and SP and lowest in the EP and this is likely due to the differences in the eyewall precipitation rates between these basins^29^. The high NI maximum precipitation rates is nonetheless surprising since the median TC intensity is relatively lower in the NI basin compared to other basins. The RA is highest in the WP, indicative of a larger region of influence of the TC, and lowest in the EP and NA, which also agrees well with the prior studies^29^ for inter-basin differences in TC size.

**Fig. 4:**
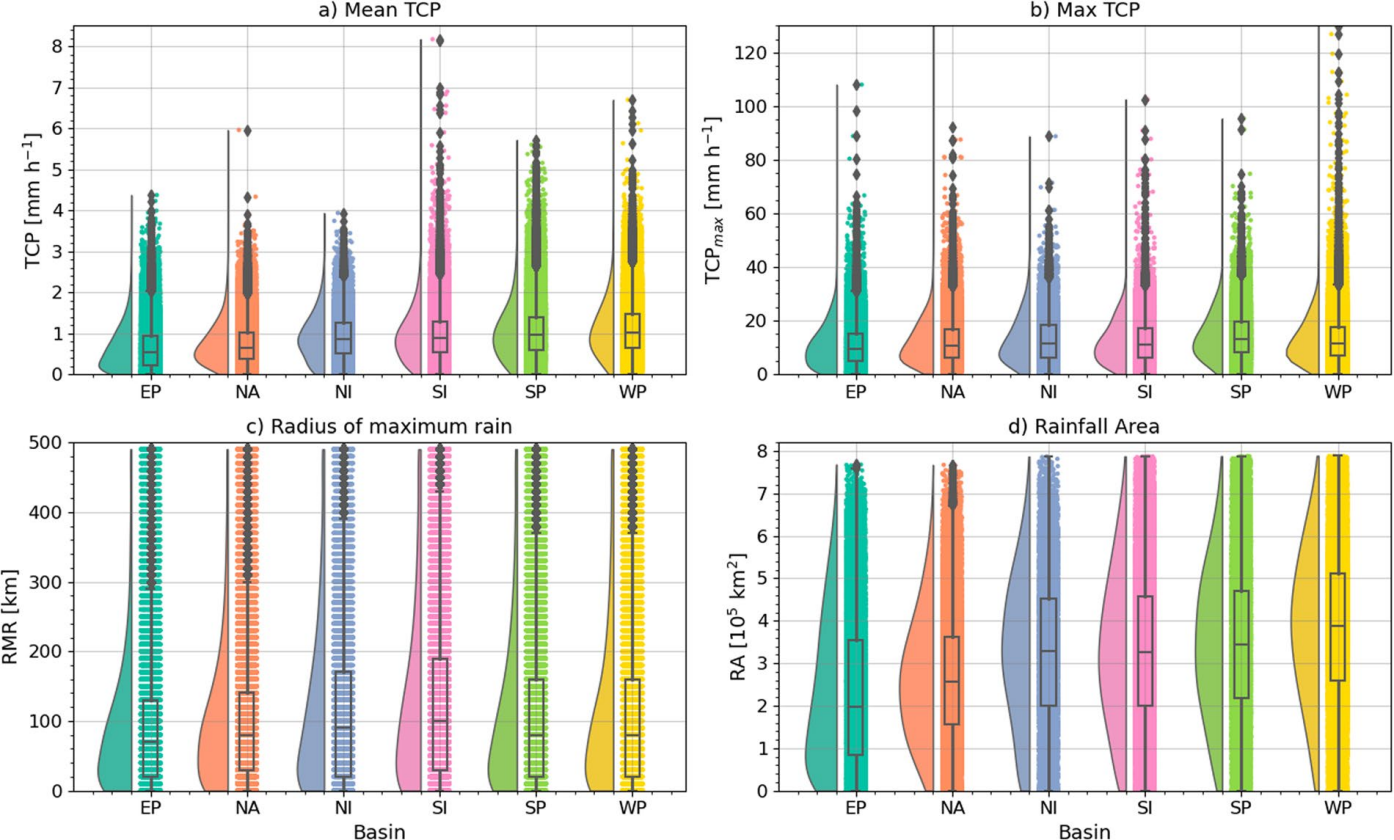
Rain cloud plot showing the violin and box plots of the distribution of (**a**) mean TCP in 500 km radii in mm h^−1^, (**b**) maximum TCP mm h^−1^, (**c**) radius of maximum rain and (**d**) rainfall area. Boxplots show the interquartile range [box], the spread [whiskers], and outliers are shown as diamonds.

Figure 4 illustrates the statistical distribution of these 4 metrics conditioned on the TC basin. While the median of the area-averaged TCP values is highest in the SP and WP, the outliers in the SI are highest among all basins. The WP TCs show the highest third quartile and outliers of TCP_*max*_ although the mean value of this metric is highest in the NI. In other words, while the average precipitation is higher in the WP, there are other aspects of the TCP distribution that are important such as the extremes, which can be obscured by any one statistic such as the mean. For instance, the mean TCP is lowest in the EP basin, but maximum rates can reach 110 mm h^−1^.

The RMR inter-basin differences (Fig. 4c) are the result of basin-wide differences in intensity which modulates the RMW as stronger storms tend to have a stronger eyewall closer to the storm centre but also in TC size which modulates the extent of the TC cloud shield and RA. Based on the RA, TCs have the smallest size in the EP as the majority of cases are consistently smaller than in basins such as WP and SP. These results are indicative of previously reported differences in the average storm size, water vapour content and intensity between basins^10,21,29^.

**Fig. 5:**
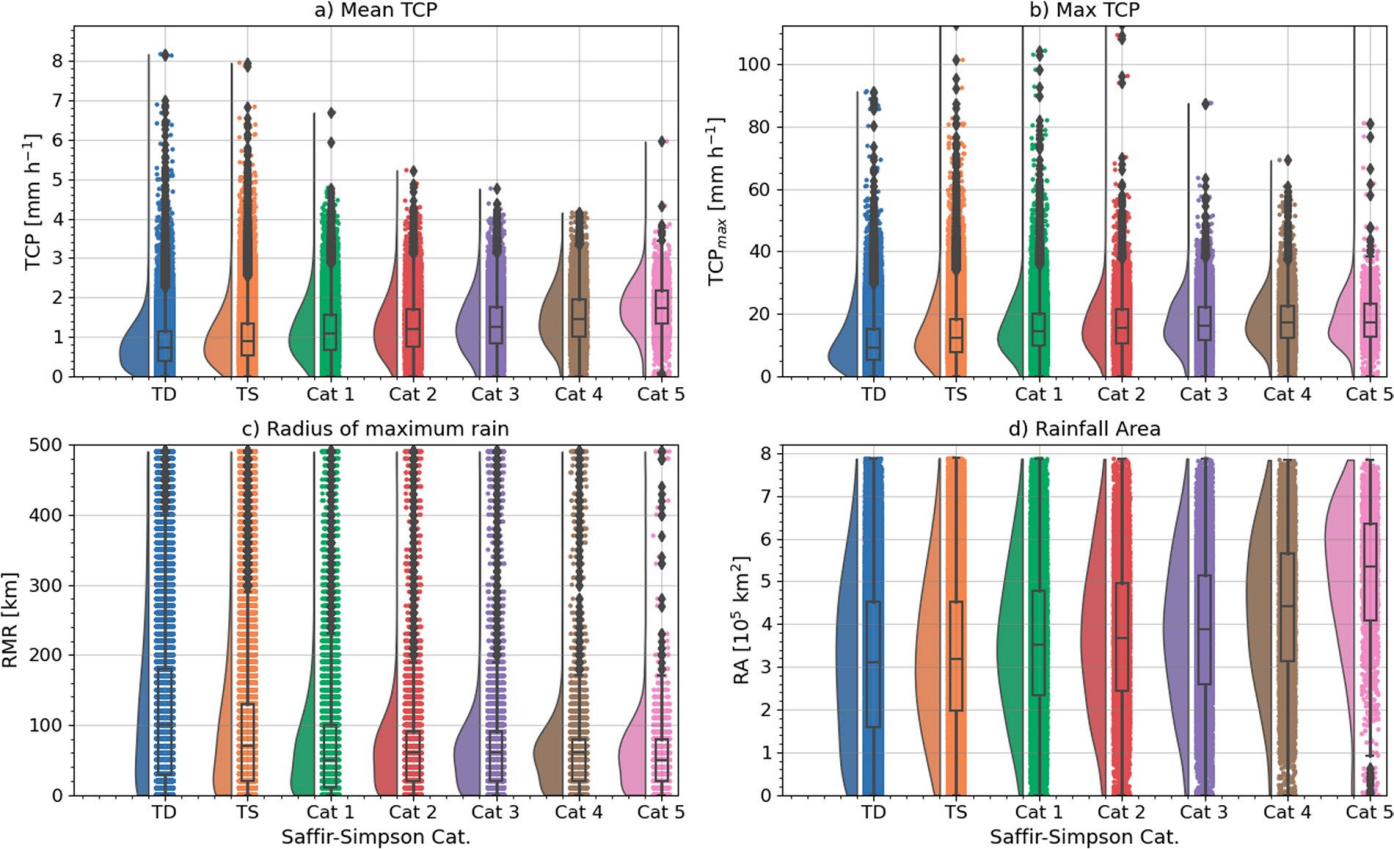
As in Fig. 4 but distributions are conditioned on the storm intensity categories per the Saffir-Simpson wind scale.

The relationship between TCP and storm intensity is summarized in Table 2 and illustrated in Fig. 5. Even though storm intensity does not explain all of the variability in TCP, the average and maximum TCP rates, as well as RA, increase with intensity whereas the RMR decreases with intensity. A stronger storm, will typically be larger and contain more total water vapour which leads to larger values of average TCP as intensity increases. However, it is apparent that weak storms in Tropical Depression or Tropical Storm status can have large values of TCP within 500 km.

**Table 2:** Mean statistics separated per TC intensity category. As in Table 1 but for different storm intensity categories based on the Saffir-Simpson wind scale.

Cat.	Percent	TCP_*mean*_ [mm h^−1^]	TCP_*max*_ [mm h^−1^]	RMR [km]	RA [10^4^ km^2^]
TD	30.2	0.81	10.9	127	31.1
TS	35.6	0.98	13.5	91.4	32.8
Cat 1	11.3	1.17	15.3	67.5	35.7
Cat 2	4.26	1.27	16.6	66.6	37.1
Cat 3	5.0	1.31	16.9	63.6	38.5
Cat 4	3.5	1.5	17.7	60.6	43.4
Cat 5	0.73	1.73	18.2	59.3	50.7

The median TCP_*max*_ rates are also larger for Cat. 4–5 storms compared with Cat. 1 and 2. However, Fig. 5b shows that storms in all categories have multiple cases where precipitation rates exceeded 50 mm h^−1^. Other factors that are not storm intensity likely modulate the maximum rates of precipitation found in TCs. Similarly, stronger storms have a smaller RMR (Fig. 5c), consistent with a stronger primary circulation and a tighter RMW which is also observed in stronger storms^17^. Stronger storms also show a larger RA (Fig. 5d), consistent with a larger secondary circulation. Even though weak storms have an average area smaller than stronger storms, TS and Cats. 1 and 2 storms frequently show very large RAs. Weak storms with large RA also emphasize that rain-related TC impacts from large RA may not be solely driven by storm intensity, e.g., the case of Hurricane Ida^49^.

The differences in the distributions depicted in Fig. 5 show that while, on average, these TCP metrics are related to the storm intensity, TCP variability is modulated by other factors. Relatively weak storms can still cause significant floods and exhibit very large precipitation rates or RAs.

### Profile dataset

In addition to the main dataset, the profile dataset provides the azimuthally averaged precipitation in 10 km bins from 0 to 500 km. This means that for each entry in the main dataset, there are 50 entries in the profile dataset where each row is the averaged precipitation in a given radial bin for a specific TC case. The radial structure of precipitation is useful to diagnose the storm structure in observed or simulated TCs^13,15,41^.

**Fig. 6:**
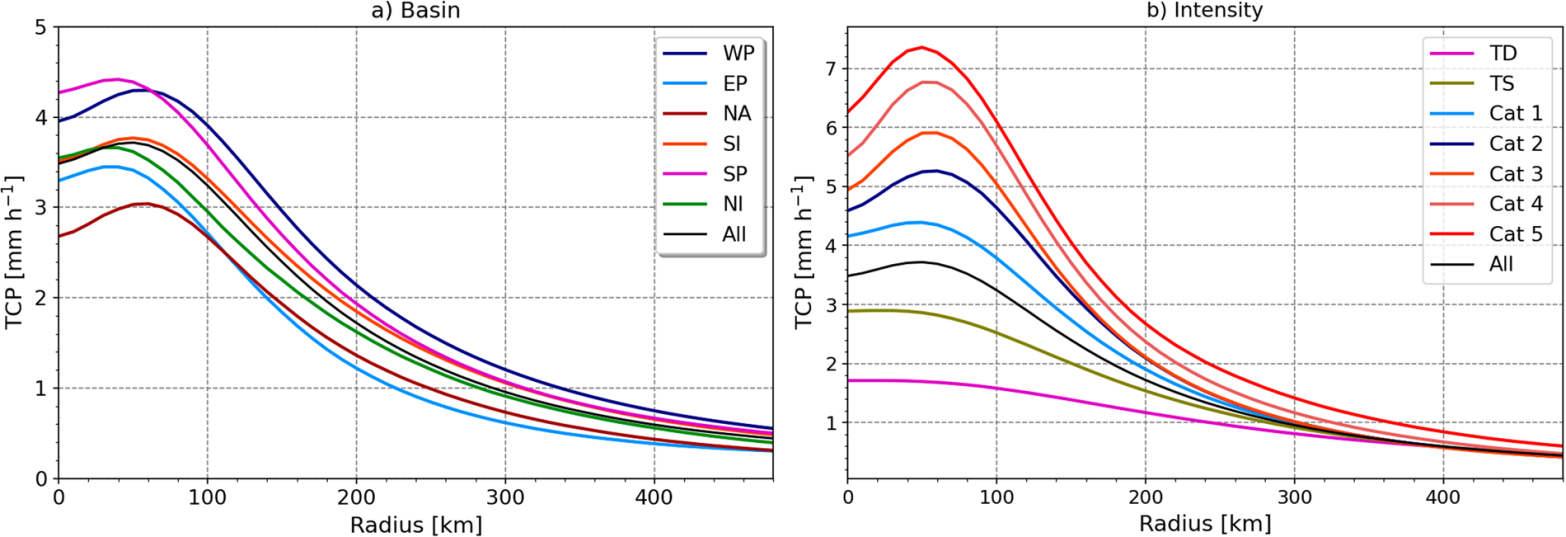
Azimuthally averaged precipitation as a function of distance from storm centre [Radius in km] for cases separated by (**a**) basin and (**b**) intensity category. In (**b**) only cases with a maximum sustained wind speed of 34 kt are considered.

Figure 6 shows the azimuthally averaged structure of TCP in all cases separated by basin and intensity category. One key feature of the radial profiles of TCP computed from MSWEP is that the inner core structure of precipitation is better resolved than in previous datasets^15,41^. The radial profile of TCP is characterized by maximum precipitation rates around 50–100 km, where the RMW is typically located, and TCP decreases both into the storm centre, where the eye is usually drier, and away from the storm centre where ascent is weaker and precipitation linked to the rain bands^17^. In MSWEP, with a 0.1° resolution, this structure is diagnosed in storms of at least TS status. This structure could not be diagnosed in previous coarser observational datasets.

Figure 6 shows that the averaged rainfall structure varies from basin to basin and as a function of storm intensity. However, the impact of storm intensity on the radial structure and peak TCP is stronger than basin-wide differences. Strong storms show a clear eyewall and RMR within 70 km of the storm centre. However, the profile of precipitation is flatter for weak storms with little variation of precipitation as a function of distance from the storm center.

These results suggest that TCs in the WP have the highest TCP rates in the inner core. While TCs in the EP have higher TCP in the inner core, the NA storms have higher precipitation rates at larger radii. However, this result requires further investigation into the mechanisms that lead to these inter-basin differences in storm structure. These results illustrate the potential use of this profile dataset to further diagnose, from a process-level understanding^13,41^, observed differences in the total precipitation associated with the azimuthally averaged structure of a TC.

## Usage Notes

The columns of the *main* dataset are:

- ISO_TIME: from IBTrACS, ISO time in Universal Time Coordinates (UTC) of the corresponding TC location (latitude, longitude);
- LAT: from IBTrACS, latitude (degrees) of the eye of the storm (LAT in IBTrACS, which is the average latitude reported by several stations);
- LON: from IBTrACS, longitude (degrees) of the eye of the storm (LON in IBTrACS, which is the average longitude reported by several stations);
- NAME: from IBTrACS, name of the storm;
- SID: from IBTrACS, unique storm identifier;
- row_id: unique row identifier to link the *main* and *profile* datasets;
- variant: from MSWEP, one of the variants available: “Past” or “NRT” (see the July 2023 Expert Developer Guidance section from the NCAR Climate Data Guide^50^)
- rain_area: rain area (km^2^) over a threshold of 0.5 mm/hr.
- area_avg_TCP: cumulative rain in a circle of 500 km from the eye averaged over rain area (mm/h/km^2^).
- lat_max_precip: latitude (degrees) of the location of the maximum precipitation;
- lon_max_precip: longitude (degrees) of the location of the maximum precipitation;
- max_precip: maximum precipitation (mm/hr) found within the circle of 500 km from the eye.
- RMR: distance (km) between the eye of the storm and the location of the maximum precipitation found (radius of maximum rain).

The radial profile dataset provides estimates of the azimuthally averaged precipitation which are often described as radial profiles of precipitation. In addition to ISO_TIME, LAT, LON, SID, row_id and variant, columns for the *radial profile* dataset include:

- bin: distance *r* (km) from the eye of the storm. A value of *r* indicates the statistic is computed between *r* to *r* + 10 km from the eye of the storm, with r = 0, 10, 20, …, 490.
- azim_avg_TCP: azimuthal average precipitation computed between *r* to *r* + 10 km from the eye of the storm.

The bin 0, or row 1, corresponds to 0–10 km range, the bin 10, or second row corresponds to 10–20 km range, up to the 490 bin, corresponding to 490–500 km range.

There are some limitations to the dataset. First, the fixed and symmetrical radius to define TCP (500 km) is a commonly used, physically-sound, proxy for the area of the TC rainfall but in several cases this threshold may be too large or too small. More complex object-tracking methodologies may be more precise in these specific cases^21^. Second, the threshold chosen for the rainfall area is arbitrary and the estimated area is sensitive to this choice. Providing statistics per quadrant (shear-relative and storm-relative) and computing rainfall area for various thresholds could improve the dataset in the future.

## Data Availability

The code to build the main and profile datasets are provided at: https://github.com/GabrielMorin1109/MSTCP-Dataset. All scripts were coded in Python and Bash and the list of dependencies can be found in the files included in the envs folder.
